# Intestinal Epithelial Toll-like Receptor 4 Deficiency Modifies the Response to the Activity-Based Anorexia Model in a Sex-Dependent Manner: A Preliminary Study

**DOI:** 10.3390/nu14173607

**Published:** 2022-08-31

**Authors:** Pauline Tirelle, Colin Salaün, Alexandre Kauffmann, Christine Bôle-Feysot, Charlène Guérin, Marion Huré, Alexis Goichon, Asma Amamou, Jonathan Breton, Jean-Luc do Rego, Pierre Déchelotte, Najate Achamrah, Moïse Coëffier

**Affiliations:** 1 Université de Rouen Normandie, INSERM UMR 1073 “Nutrition, Inflammation and Microbiota–Gut–Brain Axis”, 76183 Rouen, France; 2 Institute for Research and Innovation in Biomedicine (IRIB), Université de Rouen Normandie, 76183 Rouen, France; 3 Department of Nutrition, Rouen University Hospital, CHU Rouen, 76031 Rouen, France; 4 Université de Rouen Normandie, INSERM US51-CNRS UAR2026, Animal Behavioural Platform, SCAC-HeRacLeS “High-Tech Research Infrastructures for Life”, 76183 Rouen, France

**Keywords:** anorexia nervosa, activity-based anorexia, gut-brain axis, Toll-like receptor, behaviour

## Abstract

The role of microbiota in eating disorders has recently emerged. Previous data reported that lipopolysaccharides induce anorexia and a decrease of body weight through the activation of toll-like receptor 4 (TLR4). In the activity-based anorexia (ABA) mouse model, an increase of TLR4 expression in intestinal epithelial cells (IEC) has been described. We thus aimed to characterize the role of TLR4 in IEC in the ABA model in male and female mice. For this purpose, Vill-Cre^ERT2^-TLR4 LoxP, which are depleted for TLR4 in IEC in response to 4-OH tamoxifen, were submitted (ABA) or not (CT) to the ABA procedure that combined free access to a running wheel and progressive time-limited access to food. We thus compared CT and ABA TLR4^IEC−/−^ mice to CT and ABA TLR4^IEC+/+^ mice. In response to the ABA model, TLR4^IEC+/+^ male and female mice exhibited a body weight loss associated to a decrease of lean mass. In TLR4^IEC−/−^ male mice, body weight loss was delayed and less pronounced compared to TLR4^IEC+/+^ male mice. We did not observe a difference of body weight loss in female mice. The body composition remained unchanged between TLR4^IEC−/−^ and TLR4^IEC+/+^ mice in both sexes. In both sexes, ABA TLR4^IEC+/+^ mice exhibited an increase of food-anticipatory activity, as well as an increase of immobility time during the open field test. However, female TLR4^IEC−/−^ mice showed a decrease of the time spent at the centre and an increase of the time spent at the periphery of the open field area, whereas we did not observe differences in the male mice. In conclusion, the invalidation of TLR4 in IEC modified the response to the ABA model in a sex-dependent manner. Further studies should decipher the underlying mechanisms.

## 1. Introduction

Anorexia nervosa (AN) is an eating disorder mainly affecting young females [1]. Indeed, the AN lifetime prevalence is about 1.4% in women and 0.2% in men, affecting predominantly young women with more than 75% of diagnoses set before 22 years [2]. Associated to the high rate of mortality [3], AN is an increasing public health issue [3]. According to the fifth edition of the Diagnostic and Statistical Manual of Mental Disorders (DSM-V) [4], AN is characterized by food restriction leading to severe body weight loss (body mass index (BMI) < 18.5 kg/m^2^), an intense fear of gaining weight, and an alteration of body shape perception. 

Moreover, anxious and depressive disorders are frequently observed in AN patients [5]. The pathophysiological mechanisms of AN remain incompletely understood. It is now well-established that AN is a multifactorial disease involving environmental, psychological and biological factors [6]. During the last decade, the role of the microbiota–gut–brain axis emerged [7]. Gut microbiota dysbiosis was reported in anorectic patients [8]. Particularly, Million et al. observed a negative correlation between *E. coli* abundance and BMI [9].

Lipopolysaccharide (LPS), a component of outer membrane of Gram-negative bacteria including *E. coli*, was able to induce a decrease of food intake and of body weight in lean and obese mice [10]. In addition, the injection of low doses of LPS promoted anxiety-like behaviour with an increase of immobility time during forced swimming and tail suspension tests [11]. Interestingly, toll-like receptor 4 (TLR4), the endogenous receptors of LPS, are involved in host defence against pathogens, regulate the abundance of commensal microorganisms and maintain tissue integrity [12]. 

In a previous study, we observed that TLR4 membrane expression is increased during the commonly used activity-based anorexia (ABA) model [13] in both intestinal epithelial cells (IEC) and mucosal macrophages [14], which was associated with increased intestinal and hypothalamic inflammatory responses. However, in the hypothalamus, TLR4 expression remained unaffected. Interestingly, TLR4 knockout female mice exhibited a high mortality rate in response to ABA compared to wild-type mice [14]. 

These data suggest that TLR4 may have a dichotomic role during the ABA model by promoting an inflammatory response and anorexia but also by improving survival by mechanisms that need to be deciphered. The aim of the present study was thus to characterize the role of TLR4 expressed by intestinal epithelial cells during the ABA model by studying specific TLR4 knockout in IEC. In addition, because of the sex-dependent response to the ABA model [15], we study both male and female mice.

## 2. Materials and Methods

### 2.1. Animals

Animal experiment was approved by the regional ethical committed CENOMEXA (authorization N/05-11-12/28/11-15). Experiments were carried out in accordance with current French and European regulations. Mice were housed at 23 °C ± 1 °C with a reverse light cycle (dark phase from 10:00 a.m. to 10:00 p.m.), with free access to water and food (n = 2–5/cage). To achieve specific TLR4 invalidation in intestinal epithelial cells (IEC), C57BL/6 mice carrying a transgene with tamoxifen inducible Cre recombinase under the villin promoter (Vill-Cre^ERT2^, a kind gift from the Curie Institute, Dr Sylvie Robine, Paris, France) were crossed with floxed TLR4 mice (Jackson Laboratory, Bar Harbor, ME, USA). 

Intraperitoneal injections of 4-OH tamoxifen solution (1 mg in 100 µL, Merck, Germany) were performed daily from day (d)-4 to d0 as previously described [16]. Mice with intestinal epithelial depletion of TLR4 constituted the TLR4^IEC−/−^ group compared to unmodified group, TLR4^IEC+/+^. Mouse genotyping and TLR4 gene recombination after 4-OH tamoxifen injections were controlled as described in Supplemental Figure S1.

### 2.2. Activity-Based Anorexia Model

The ABA procedure started at d1, and mice were individually placed in either standard cage (CT-TLR4^IEC+/+^ and CT-TLR4^IEC−/−^ groups) (n = 6/5 for male and female) or cages equipped with an activity wheel (ABA-TLR4^IEC+/+^ and ABA-TLR4^IEC−/−^ groups) (ABA-TLR4^IEC+/+^ n = 6/5 for male and female) (ABA-TLR4^IEC−/−^ n = 7/6 for male and female). The ABA model was performed as previously described [17]. 

Briefly, the ABA model combines a free running wheel access and a progressively limited food access from 6 h/day to 3 h/day (Supplemental Figure S2). Food was given at the beginning of dark phase (10:00 a.m.). Animals had free access to water. Running wheel activity was continuously recorded with Activity Wheel software (IntelliBio, Seichamps, France). Food-anticipatory activity was measured during the 3 h before the access to food as previously described [15]. In addition, food intake and body weight were measured daily. If body weight loss was higher than 20% on three consecutive days, the animals were killed in accordance with the ethical procedure.

### 2.3. Open Filed Test

In order to assess anxiety-like behaviour, open field tests were performed at d3 and d17, during the dark phase and after the feeding period (at 01:00 p.m.). The data were collected with Fusion Software (Omnitech Electronics Inc., Columbus, OH, USA).

### 2.4. Body Composition Assessment

Following the open field test, body composition was measured on vigil animals at d3 and d17 using a Minispec LF110 (Brucker, Wissembourg, France), a fast-nuclear magnetic resonance method to evaluate fat and lean mass.

### 2.5. Euthanasia and Sample Collection

Mice were anesthetized by the intraperitoneal injection of Ketamine/Xylazine solution (100 and 10 mg/kg of body weight, respectively). Blood samples were collected by puncture in the abdominal aorta, and plasma samples were then collected after centrifugation (3000× *g*; 20 min; 4 °C). The hypothalamus was removed and immediately frozen in liquid nitrogen. The colon was collected and washed with ice-cold PBS, and 1 cm sections were performed and immediately frozen in liquid nitrogen. All samples were then stored at −80 °C until analysis.

### 2.6. Evaluation of Plasma Leptin, Adiponectin and Corticosterone

Plasma leptin (R&D system, Minneapolis, MN, USA), adiponectin (Invitrogen, Carlsbad, CA, USA) and corticosterone (Abnova, Ann Arbor, MI, USA) levels were assessed using an enzyme-like immunosorbent assay according to the manufacturer′s instructions.

### 2.7. RNA Extraction and RT-qPCR

The total RNAs from hypothalamus and colonic mucosa were extracted by Trizol method (Invitrogen) following the manufacturer′s guidelines. After DNAse treatment (Promega, Charbonnières-les-Bains, France), RNAs were reverse-transcribed as previously described [18]. Then, qPCR was performed by using SYBRGreen technology on a Bio-Rad CFX96 real-time PCR system (Bio-Rad Laboratories, Marnes la Coquette, France). RP18S gene was used as housekeeping gene. Specific primer sequences of genes of interest are displayed in Table 1. The relative concentration was obtained by conversion of the cycle threshold on the concentration value by using a standard curve.

### 2.8. Statistical Analysis

The data were analysed using GraphPad Prism 6.0 software (GraphPad Software Inc., San Diego, CA, USA) and expressed as the mean ± standard error to mean. Values were compared by repeated two-way ANOVA (time × group, for body weight and food-anticipatory activity data) or two-way ANOVA (ABA × TLR4^IEC^) followed by Bonferroni post hoc tests, as appropriate. The results were considered significant when the *p*-value was lower than 0.05. All exact *p*-values are displayed in Supplemental Table S1.

## 3. Results

### 3.1. Effects of Intestinal Epithelial TLR4 Knockout on Body Weight, Body Composition and Food Intake in Response to ABA Model

**Figure 1:**
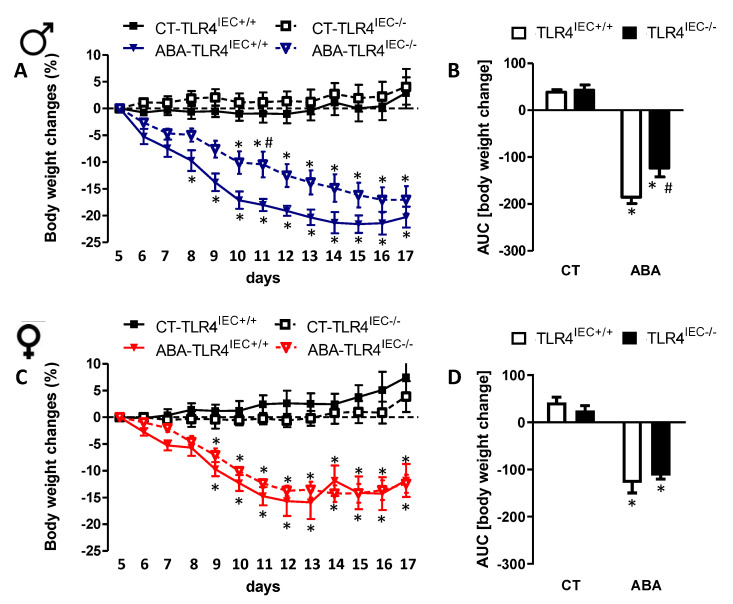
Body weight change in male and female mice. Body weight change from d5 to d17 (**A**), in control (CT-TLR4^IEC+/+^, closed squares), CT-TLR4^IEC−/−^ (open squares), ABA-TLR4^IEC+/+^ (closed triangles) and ABA-TLR4^IEC−/−^ (open triangles) male mice. Area under cover (**B**) between at the left CT TLR4^IEC+/+^ (open bars) and TLR4^IEC−/−^ (closed bars) and at the right ABA TLR4^IEC+/+^ (open bars), TLR4^IEC−/−^ (closed bars) male mice. Body weight change from d5 to d17 (**C**), in control (CT-TLR4^IEC+/+^, closed squares), CT-TLR4^IEC−/−^ (open squares), ABA-TLR4^IEC+/+^ (closed triangles) and ABA-TLR4^IEC−/−^ (open triangles) female mice. Area under cover (**D**) between at the left CT TLR4^IEC+/+^ (open bars) and TLR4^IEC−/−^ (closed bars) and at the right ABA TLR4^IEC+/+^ (open bars), TLR4^IEC−/−^ (closed bars) female mice. The results of the Bonferroni post hoc tests are shown: *, *p* < 0.05 vs. CT; #, *p* < 0.05 vs. TLR4^IEC+/+^.

**Figure 2:**
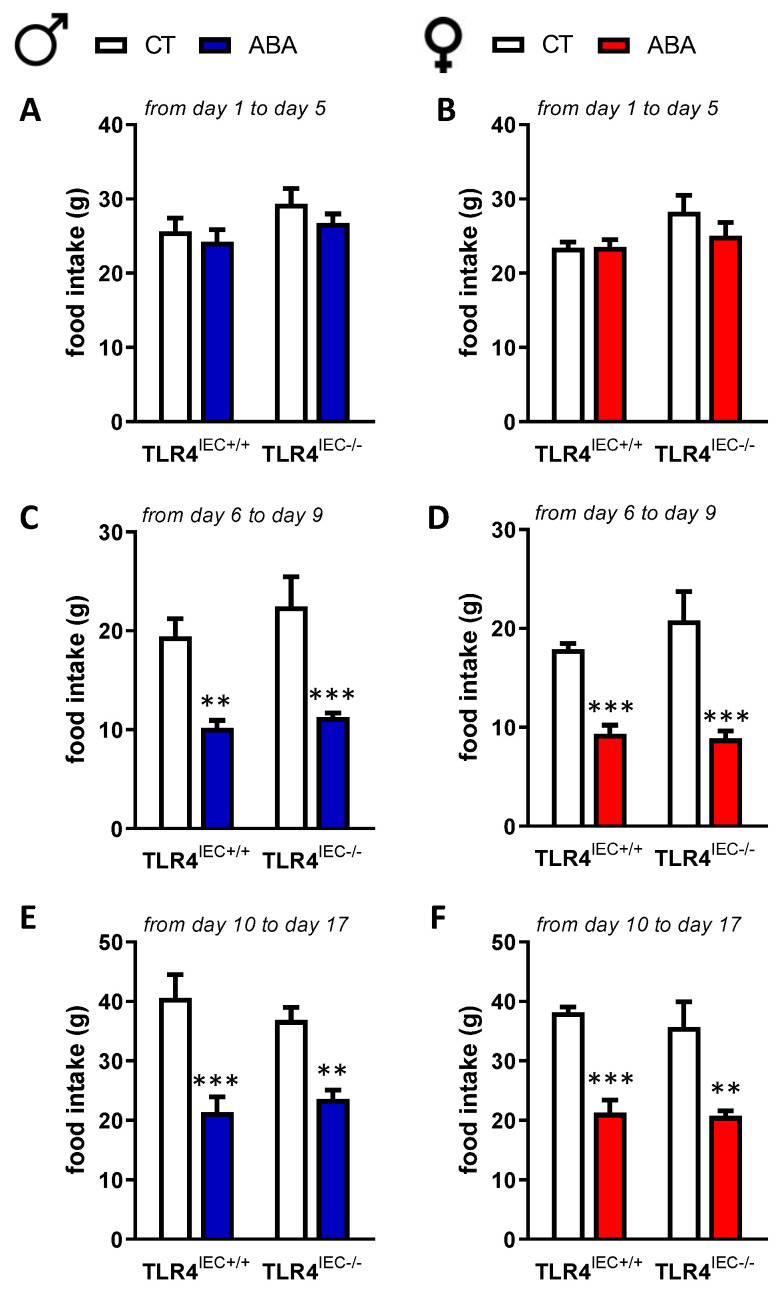
Food intake in male and female mice. Food intake was measured during the adaptation phase (from day 1 to day 5, **A**,**B**), the progressive limitation of food access (from day 6 to day 9, **C**,**D**) and during the 3-h limited food access (from day 10 to day 17, **E**,**F**) in male TLR4^IEC+/+^ and TLR4^IEC−/−^ control (CT, open bars) and ABA (blue bars) mice or in female TLR4^IEC+/+^ and TLR4^IEC−/−^ control (CT, open bars) and ABA (reds bars) mice. The results of the Bonferroni post hoc tests are shown: **, *p* < 0.01 and ***, *p* < 0.001 vs. CT.

During the experiment, control (TLR4^IEC+/+^) and TLR4^IEC−/−^ mice exhibited similar body weight (Figure 1) and similar food intake (Figure 2). Before the limitation of food access, body composition was similar between the groups (Supplemental Figure S3). The limitation of food access time induced a body weight loss in all ABA groups (Figure 1). However, in male mice, the kinetics of body weight loss were different between ABA TLR4^IEC−/−^ and ABA TLR4^IEC+/+^ mice. Indeed, ABA TLR4^IEC+/+^ mice lost significant weight from day 8 to day 17 compared to the control TLR4^IEC+/+^ mice, while ABA TLR4^IEC−/−^ mice lost body weight from day 10 (Figure 1A). 

In addition, the body weight loss was significantly lower in ABA TLR4^IEC−/−^ mice compared to ABA TLR4^IEC+/+^ mice at day 11. To provide evidence of different kinetics of body weight loss, we analysed the area under the curve (AUC) showing that AUC of body weight loss was lower in ABA TLR4^IEC−/−^ mice compared to ABA TLR4^IEC+/+^ mice (Figure 1B). Interestingly, we did not observe a similar pattern in female mice. Indeed, female ABA mice showed the same body weight loss kinetics whatever TLR4 genetic background (Figure 1C,D). 

**Figure 3:**
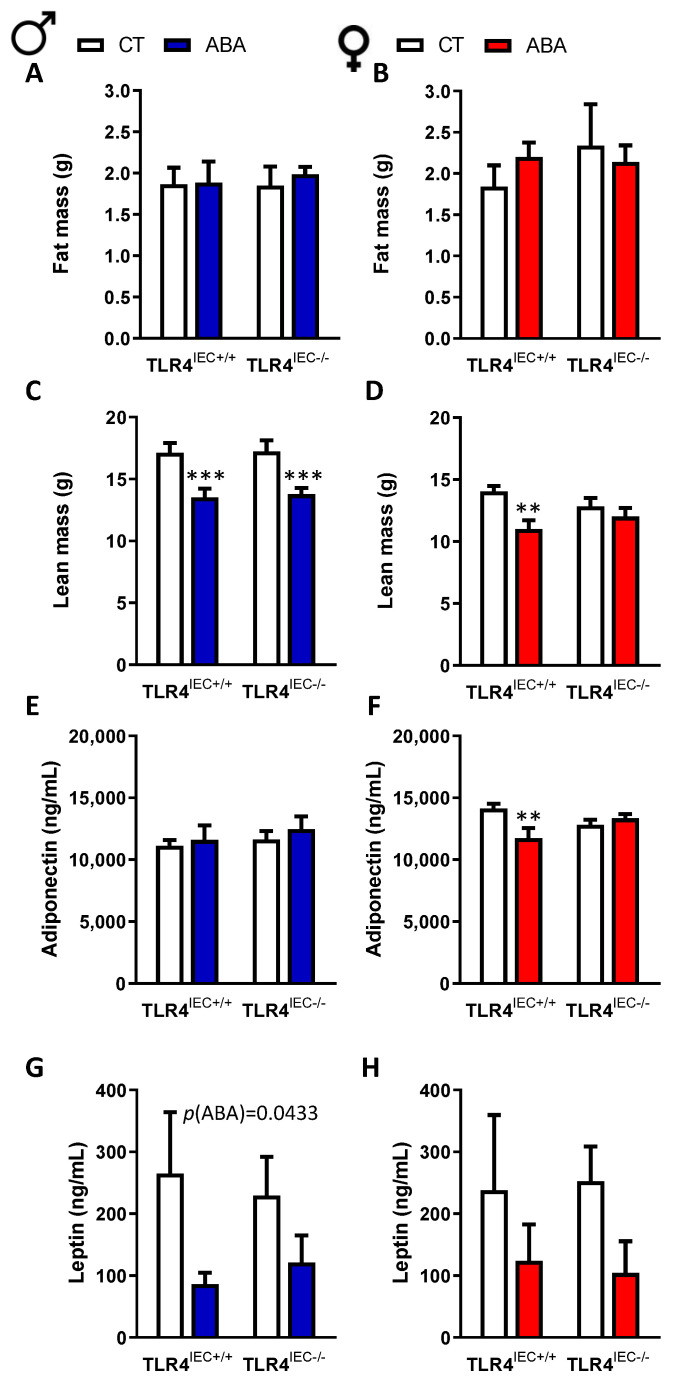
Body composition at day 17 and the plasma leptin and adiponectin levels in male and female mice. Fat (**A**) and lean mass (**C**) in TLR4^IEC+/+^ and TLR4^IEC−/−^ CT (open bars) and ABA (blue bars) male mice. Fat (**B**) and lean mass (**D**) between TLR4^IEC+/+^ and TLR4^IEC−/−^ CT (open bars) and ABA (reds bars) female mice. Adiponectin (**E**) and leptin (**G**) plasma level in TLR4^IEC+/+^ and TLR4^IEC−/−^ CT (open bars) and ABA (blue bars) male mice. Adiponectin (**F**) and leptin (**H**) plasma level in TLR4^IEC+/+^ and TLR4^IEC−/−^ CT (open bars) and ABA (reds bars) female mice. The results of the Bonferroni post hoc tests are shown: **, *p* < 0.01 and ***, *p* < 0.001 vs. CT; *p* < 0.05 vs. TLR4^IEC+/+^.

Body composition was mainly affected by ABA procedure with a decrease of lean mass in both male and female mice (Figure 3). TLR4 invalidation in IEC did not affect markedly body composition, even if the difference between ABA TLR4^IEC−/−^ and control TLR4^IEC−/−^ did not reach significance. As shown in the Figure 2, food intake was decreased during the ABA procedure both in male and female mice; however, TLR4 invalidation in IEC did not affect it whatever the studied period (adaptation phase, progressive limited access to food or limited access to food). Concerning the adipokine plasma levels, adiponectin was not modified in male mice. 

In contrast, adiponectin was reduced in female ABA TLR4^IEC+/+^ compared to the controls as it was not present in female ABA TLR4^IEC−/−^ (Figure 3F). We observed a reduction of the leptin level in ABA mice, even if the difference did not reach significance in female mice without any effect of TLR4 invalidation (Figure 3G,H).

### 3.2. Effects of Intestinal Epithelial TLR4 Knockout on Behavioural Response

**Figure 4:**
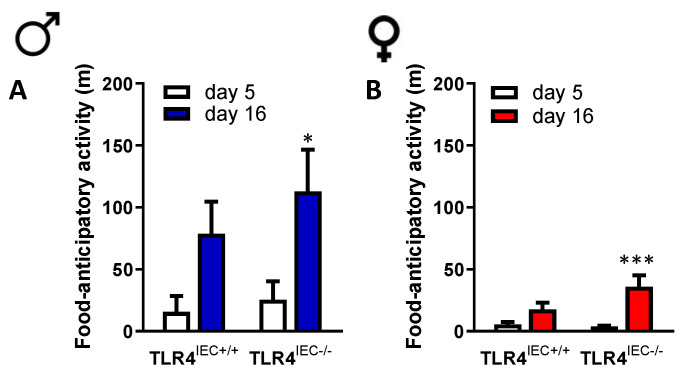
Food-anticipatory activity between d5 and d16 in ABA TLR4^IEC+/+^ and TLR4^IEC−/−^ in male and female mice. Food-anticipatory activity between d5 (open bars) and d16 (blue bars) in ABA TLR4^IEC+/+^ and TLR4^IEC−/−^ male mice (**A**). Food-anticipatory activity between d5 (open bars) and d16 (red bars) in ABA TLR4^IEC+/+^ and TLR4^IEC−/−^ female mice (**B**). The results of the Bonferroni post hoc tests are shown: *, *p* < 0.05 and ***, *p* < 0.001 vs. CT.

Before the limitation of food access, we did not observe differences in the behavioural responses between the groups (Supplemental Figure S4). After the beginning of the limitation of food access, we observed a trend of an increase of wheel activity both in male and female mice that was not affected by intestinal epithelial TLR4 invalidation (Supplemental Figure S5). During the ABA procedure, food-anticipatory activity increased in both male and female mice between day 5 and day 16 (Figure 4). 

**Figure 5:**
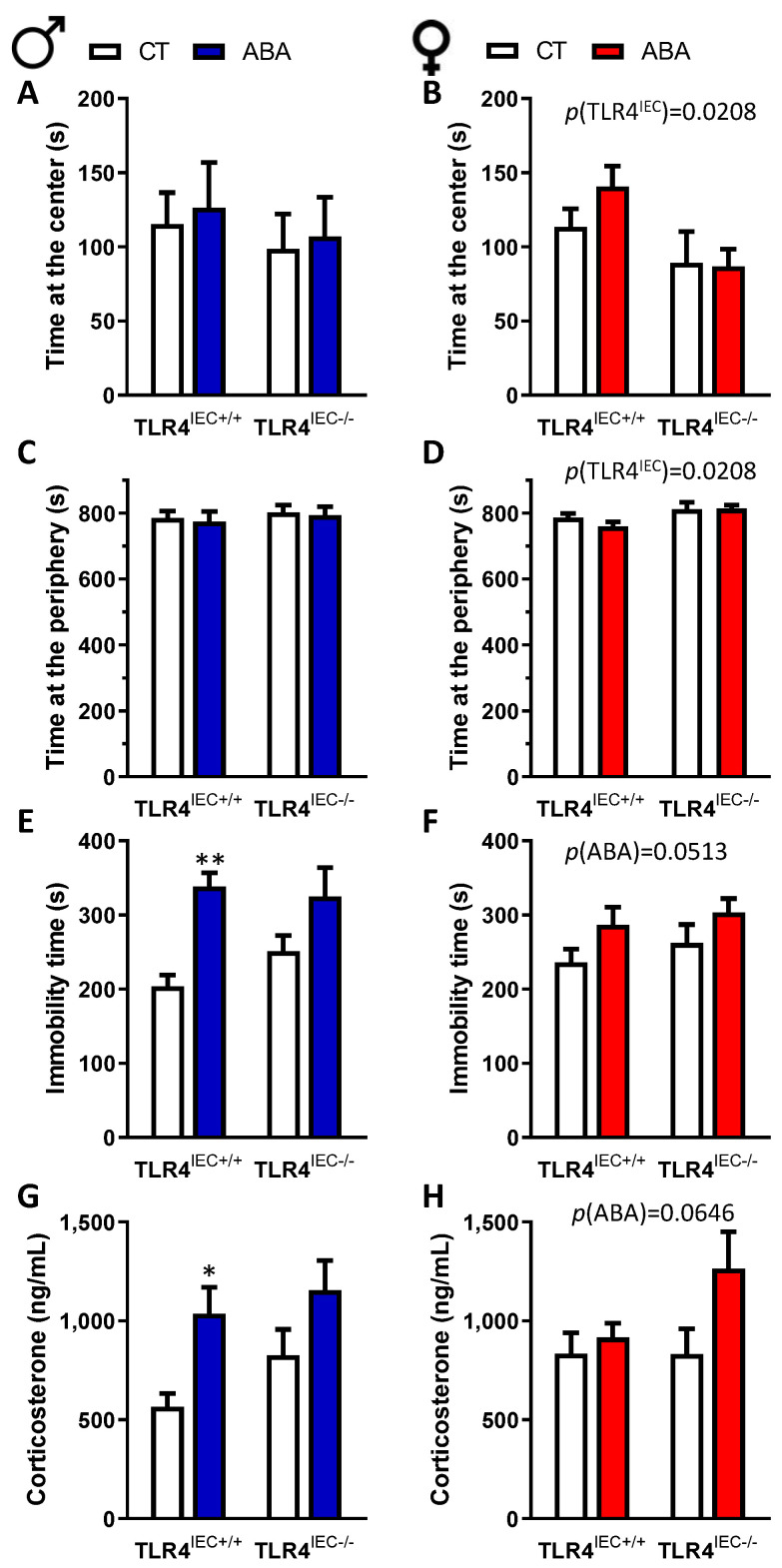
Open field test at d17 and corticosterone plasma levels in male and female mice. Time at the centre (**A**), the periphery (**C**) and immobility time (**E**) during open field test and corticosterone plasma level (**G**) between TLR4^IEC+/+^ and TLR4^IEC−/−^ in CT (open bars) and ABA (blue bars) male mice. Time at the centre (**B**), the periphery (**D**) and immobility time (**F**) during open field test and corticosterone plasma level (**H**) between TLR4^IEC+/+^ and TLR4^IEC−/−^ in CT (open bars) and ABA (reds bars) female mice. The results of the Bonferroni post hoc tests are shown: *, *p* < 0.05 and **, *p* < 0.001 vs. CT; *p* < 0.05 vs. TLR4^IEC+/+^.

However, in females, ABA TLR4^IEC−/−^ exhibited a trend for an increase of food-anticipatory activity compared to ABA TLR4^IEC+/+^ but the difference did not reach significance (Figure 4B, *p* = 0.092). In the same way, only female mice showed altered behavioural response during the open field test according to the TLR4 genetic background. Indeed, although time spent at the centre or at the periphery remained unchanged in male mice (Figure 5A,C), female TLR4^IEC−/−^ mice exhibited lower time spent at the centre and more time spent at the periphery (Figure 5B,D) both in control and ABA mice. The distance travelled at the centre or at the periphery remained unaffected both in female and male mice (data not shown). 

In male mice, immobility time during the open field test was increased in response to ABA (two-way ANOVA *p*(ABA) = 0.0009). However, post-tests revealed that immobility time was significantly increased in ABA TLR4^IEC+/+^ mice compared to the controls but the difference did not reach significance in ABA TLR4^IEC−/−^ mice (Figure 5E). A similar pattern was observed for plasma corticosterone in male mice (Figure 5G). In female mice, a trend for an increase of immobility time in ABA mice was observed (two-way ANOVA *p*(ABA) = 0.0513), as well as for an increased corticosterone level (Figure 5H, two-way ANOVA *p*(ABA) = 0.0646).

### 3.3. Effects of Intestinal Epithelial TLR4 Knockout on the Hypothalamic Response to ABA

**Figure 6:**
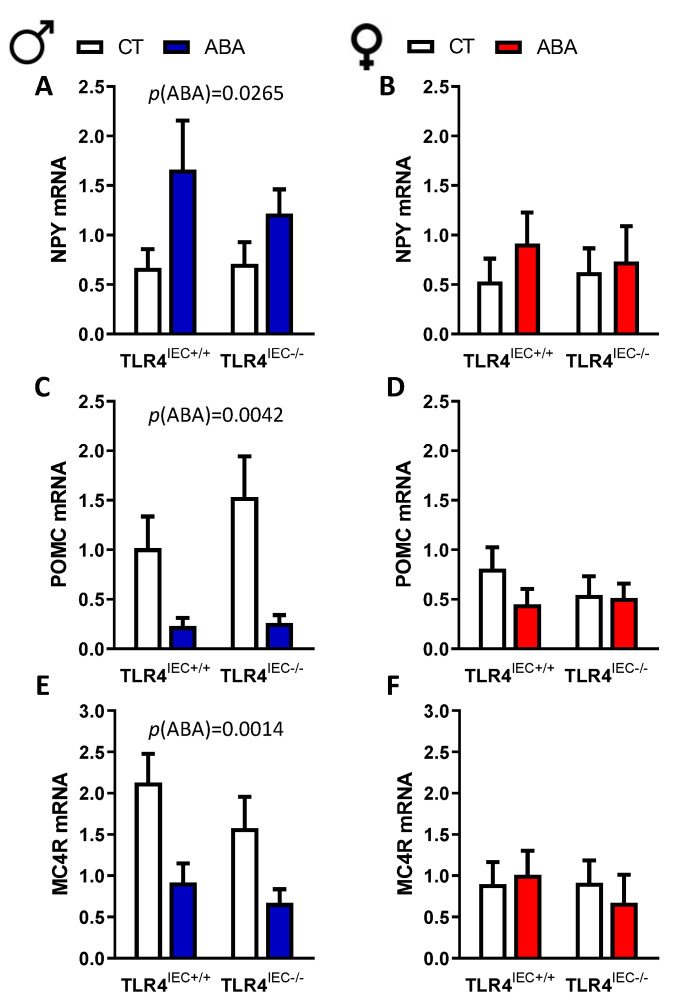
Hypothalamic neuropeptide Y (NPY), pro-opiomelanocortin (POMC) and melanocortin 4 receptor (MC4R) mRNA expression in male and female. NPY (**A**), POMC (**C**) and MC4R (**E**) hypothalamic mRNA expression between TLR4^IEC+/+^ and TLR4^IEC−/−^ in CT (open bars) and ABA (blue bars) male mice. Female mice NPY (**B**), POMC (**D**) and MC4R (**F**) hypothalamic mRNA expression between TLR4^IEC+/+^ and TLR4^IEC−/−^ in CT (open bars) and ABA (reds bars) female mice.

In the hypothalamus, male mice showed an adaptive response to food restriction with an increase of neuropeptide Y (NPY) mRNA level and a reduction of pro-opiomelanocortin (POMC) and melanocortin 4 receptor (MC4R) mRNA levels in ABA mice compared to the controls (Figure 6). However, TLR4 invalidation in IEC did not affect this response. In female mice, we did not observe significant modifications of those parameters. In addition, we also evaluated hypothalamic TLR4, Brain-Derived Neurotrophic Factor (BDNF) and Interleukin (IL-6) mRNA levels that were unchanged both in male and female mice (data not shown).

## 4. Discussion

Anorexia nervosa, an eating disorder with female predominance [3], is a multifactorial disease involving environmental, psychologic and biologic factors with an aetiology remaining poorly understood. During the last decade, the involvement of gut microbiota has emerged in the regulation of feeding behaviour and mood disorders [8]. A gut microbiota dysbiosis has been described in anorectic patients [19] and in mice submitted to the ABA model [20]. It is well established that endotoxins of the outer membrane of gram-negative bacteria, LPS, induce a decrease of food intake and body weight [21] through the activation of TLR4 [22]. 

TLR4 activation can lead to two distinct signalling cascades: the MyD88-dependent pathway responsible for the expression of pro-inflammatory cytokines and the independent MyD88 pathway mediating the expression of interferons [23]. Interestingly, Belmonte et al. reported an increase of TLR4 expression in intestinal epithelial cells during ABA model [14], suggesting that intestinal expression of TLR4 may contribute to ABA response. In the present study, we show for the first time that specific knockout of TLR4 in intestinal epithelial cells affects the response to the ABA model in a sex-dependent manner.

As previously reported [15,24], we observed a more severe body weight loss in response to the ABA model in males compared to females. Interestingly, we also observed a sex-dependent response to the ABA model according to the expression of intestinal epithelial TLR4. Indeed, only in males, TLR4^IEC−/−^ mice exhibited a lower body weight loss than TLR4^IEC+/+^ mice in response to the ABA model. In female mice, we did not observe any difference. Regarding body composition, we observed a decrease of lean mass in both male and female mice in response to the ABA model as previously described [25]. However, the TLR4 depletion in IEC did not induce modification of the body composition. 

Ogimoto et al. showed that pooled male and female mice invalidated for the TLR4-MyD88 pathway at the whole body level (Myd88^−/−^ mice) exhibited a limitation of body weight loss in response to LPS administration, as well as a restored food intake, compared to wild-type LPS-treated mice. However, in our study, only IEC were knockout for TLR4, which may contribute to explaining the absence of modification in the food intake between TLR4^IEC+/+^ and TLR4^IEC−/−^ mice. In the context of diet-induced obesity, Everard et al. reported that the invalidation of MyD88 in IEC protects against diet-induced obesity through an increase of energy expenditure without food intake alteration in male mice [26]. 

However, in the present study, we did not evaluate energy expenditure. We observed that ABA mice had an increase in food-anticipatory activity as previously described [24] but ABA-TLR4^IEC−/−^ mice did not exhibit a significant modification. Further studies should evaluate the effects of TLR4 knockout in IEC on energy expenditure according with the dark/light phases in response to the ABA model. To our knowledge, there is no previous study reporting a sex-dependent body weight change in intestinal TLR4 knockout. Previous studies reported gut dysbiosis in anorectic patients [8,19]. 

Similarly, in response to the ABA model, both rats [27] and mice [20] exhibit gut microbiota alterations; however, these data have only been obtained in males. Everard et al. also showed that MyD88 invalidation in IEC alters gut microbiota ecology [26]. It should thus be of interest to evaluate the effects of TLR4 deficiency in IEC on the gut microbiota during the ABA model in both female and male mice.

Anxiety-like behaviour is a frequent comorbidity during AN [5,28], and the role of the microbiota–gut–brain axis has been suggested [29]. Both female and male ABA mice exhibited an increase of immobility time in response to the ABA model. In male mice, we did not observe major modifications of behaviour evaluated in the open field test according with TLR4 expression. In contrast, in females, TLR4^IEC−/−^ mice show a decrease of time spent at the centre and an increase of the time spent at the periphery of the open field area in both CT and ABA groups. It is well established that TLR4 may contribute to anxiety-like behaviour [30] through central and/or peripheral actions. Thereby, TLR4 knockout mice show an increase of anxiety-like behaviour and a decrease of social interaction compared to the control mice [31]. 

Moreover, in a murine model of Alzheimer disease, MyD88^−/−^ mice exhibit an increase of anxiety-like behaviour during the elevated plus maze test [32]. Recently, the role of microbiota–gut–brain axis in anxiety-like behaviour has been suggested [33]. Fields et al. showed that oral administration of LPS induced anxiety-like behaviour in both male and female mice [34]. Most interestingly, the authors also reported that treatment with naloxone, which blocks the TLR4-TRIF pathway, had opposing behavioural effects in male and female LPS-treated mice [34]. The sex differences in behaviour responses may be explained by sex differences in the cytokine responses to TLR4 activation [35].

In the present study, we included control ad libitum and ABA groups. It would also be interesting to evaluate the effects of IEC-specific TLR4 invalidation in starved animals (limited food access with no running wheel) or in animals with access to a running wheel without food restriction to distinguish the effects of activity and starvation as performed in previous studies [36,37]. In addition, we evaluated anxiety-like behaviour at day 17, a late time point associated with a stable body weight and no difference between the groups. It would be of interest to evaluate anxiety-like behaviour at an earlier stage of the ABA model when body weight loss occurs and when TLR4^IEC−/−^ and TLR4^IEC+/+^ ABA mice exhibit body weight differences, e.g., day 11 in male mice. Further experiments should be done to investigate these points.

## 5. Conclusions

In conclusion, we report, for the first time, that TLR4 invalidation in IEC during the ABA model induced a sex-dependent response: a delayed body weight loss in males and an increase of anxiety-like behaviour in females. Further studies should be done to better understand the underlying mechanisms.

## Figures and Tables

**Table 1:** Primer sequences.

Gene	Forward Primer	Reverse Primer
*RPS18*	TGCGAGTACTCAACACCAACA	TTCCTCAACACCACATGAGC
*GAPDH*	ATCACTGCCACTCAGAAGA	TCACTGCCACTCAGAAGA
*NPY*	CTGCGACACTACATCAATCT	CTTCAAGCCTTGTTCTGG
*POMC*	CCTCCTGCTTCAGACCTCCA	GGCTGTTCATCTCCGTTGC
*MC4R*	TCTCTATGTCCACATGTTCCTG	GGGGCCCAGCAGACAACAAAG
*BDNF*	TGTGACAGTATTAGCGAGTGG	TACGATTGGGTAGTTCGGCATT
*TLR4*	AGATCTGAGCTTCAACCCCTTG	AGAGGTGGTGTAAGCCATGC
*IL-6*	TAGTCCTTCCTACCCCAATTTCC	TTGGTCCTTAGCCACTCCTTC
*For genotyping*		
*Villin-Cre^ERT2^*	CAAGCCTGGCTCGACGGCC	CGCGAACATCTTCAGGTTCT
*TLR4LoxP*	TGACCACCATATTGCCTATAC	TGATGGTGTGAGCAGGAGAG
*To control DNA recombination*		
*TLR4*	GAACCTAGTACATGTGGATCTTTCTTATAACT	GTCTTGAATGAAGTCAATTGGGTTCA
*Cre Activity*	TGACCACCCATATTGCCTATAC	CCTCTTCTGTGCTATCTGGC

## Data Availability

Not applicable.

## Supplementary Materials

The following are available online at https://www.mdpi.com/article/10.3390/nu14173607/s1, Table S1: Exact p values for two-way ANOVA; Figure S1: Mouse genotyping PCR; Figure S2: Timeline of experimental protocol; Figure S3: Body composition at day 3 in male and female mice; Figure S4: Open field test at d3 in male and female mice; Figure S5: Total wheel activity in male and female ABA mice.
