# Supplementary material for: Intestinal Epithelial Toll-like Receptor 4 Deficiency Modifies the Response to the Activity-Based Anorexia Model in a Sex-Dependent Manner: A Preliminary Study
Source: Nutrients. 2022 Aug 31;14(17):3607. doi: 10.3390/nu14173607 (PMC9460860; doi:10.3390/nu14173607)
Supplement: Supplementary file 1 [file nutrients-14-03607-s001.zip › nutrients-1828527-supplementary.pdf]

Supplemental Table S1: Exact p values for two-way ANOVA

	Figure	Exact p-values for two-way ANOVA		
Body weight change				
For male mice	Fig. 1B	**p(ABA)<0.0001**	**p($TLR4^{IEC}$)=0.0263**	p(Int)=0.0511
For female mice	Fig. 1D	**p(ABA)<0.0001**	p($TLR4^{IEC}$)=0.9394	p(Int)=0.2526
Food intake (day 1 – day 5)				
For male mice	Fig. 2A	p(ABA)=0.2464	p($TLR4^{IEC}$)=0.0772	p(Int)=0.7267
For female mice	Fig. 2B	p(ABA)=0.3412	p($TLR4^{IEC}$)=0.0625	p(Int)=0.3051
Food intake (day 6 – day 9)				
For male mice	Fig. 2C	**p(ABA)<0.0001**	p($TLR4^{IEC}$)=0.2538	p(Int)=0.5822
For female mice	Fig. 2D	**p(ABA)<0.0001**	p($TLR4^{IEC}$)=0.4326	p(Int)=0.2861
Food intake (day 10 – day 17)				
For male mice	Fig. 2E	**p(ABA)<0.0001**	p($TLR4^{IEC}$)=0.7755	p(Int)=0.2702
For female mice	Fig. 2F	**p(ABA)<0.0001**	p($TLR4^{IEC}$)=0.5384	p(Int)=0.6897
Body composition at day 3				
Fat mass, For male mice	Fig. S3A	p(ABA)=0.3388	p($TLR4^{IEC}$)=0.9418	p(Int)=0.9418
Fat mass, For female mice	Fig. S3B	p(ABA)>0.9999	p($TLR4^{IEC}$)=0.8081	p(Int)=0.6281
Lean mass, For male mice	Fig. S3C	p(ABA)=0.6220	p($TLR4^{IEC}$)=0.8790	p(Int)=0.7868
Lean mass, For female mice	Fig. S3D	p(ABA)=0.8451	p($TLR4^{IEC}$)=0.7430	p(Int)=0.5056
Body composition at day 17				
Fat mass, For male mice	Fig. 3A	p(ABA)=0.7032	p($TLR4^{IEC}$)=0.8301	p(Int)=0.7658
Fat mass, For female mice	Fig. 3B	p(ABA)=0.8010	p($TLR4^{IEC}$)=0.4911	p(Int)=0.3830
Lean mass, For male mice	Fig. 3C	**p(ABA)<0.0001**	p($TLR4^{IEC}$)=0.8037	p(Int)=0.9086
Lean mass, For female mice	Fig. 3D	**p(ABA)=0.0082**	p($TLR4^{IEC}$)=0.8657	p(Int)=0.1021
Adiponectin				
For male mice	Fig. 3E	p(ABA)=0.4842	p($TLR4^{IEC}$)=0.4574	p(Int)=0.8454
For female mice	Fig. 3F	p(ABA)=0.0776	p($TLR4^{IEC}$)=0.7718	**p(Int)=0.0099**
Leptin				
For male mice	Fig. 3G	**p(ABA)=0.0433**	p($TLR4^{IEC}$)=0.9989	p(Int)=0.5987
For female mice	Fig. 3H	p(ABA)=0.1167	p($TLR4^{IEC}$)=0.9763	p(Int)=0.8300
Food anticipatory activity				
For male mice	Fig. 4A	**p(Time)=0.0039**	p($TLR4^{IEC}$)=0.3436	p(Int)=0.5957

For female mice	Fig. 4B	**p(Time)=0.0005**	p($TLR4^{IEC}$)=0.1164	p(Int)=0.0597
<u>Open field test at day 3</u>				
Time at the center, For male mice	Fig. S4A	p(ABA)=0.4869	p($TLR4^{IEC}$)=0.7126	p(Int)=0.2866
Time at the center, For female mice	Fig. S4B	p(ABA)=0.7744	p($TLR4^{IEC}$)=0.9882	p(Int)=0.5856
Time at the periphery, For male mice	Fig. S4C	p(ABA)=0.4869	p($TLR4^{IEC}$)=0.7126	p(Int)=0.2866
Time at the periphery, For female mice	Fig. S4D	p(ABA)=0.7744	p($TLR4^{IEC}$)=0.9882	p(Int)=0.5856
Immobility time, For male mice	Fig. S4E	p(ABA)=0.4197	p($TLR4^{IEC}$)=0.9746	p(Int)=0.5422
Immobility time, For female mice	Fig. S4F	p(ABA)=0.4465	p($TLR4^{IEC}$)=0.6973	p(Int)=0.2121
<u>Open field test at day 17</u>				
Time at the center, For male mice	Fig. 5A	p(ABA)=0.7120	p($TLR4^{IEC}$)=0.4931	p(Int)=0.9579
Time at the center, For female mice	Fig. 5B	p(ABA)=0.4254	**p($TLR4^{IEC}$)=0.0208**	p(Int)=0.3433
Time at the periphery, For male mice	Fig. 5C	p(ABA)=0.7120	p($TLR4^{IEC}$)=0.4931	p(Int)=0.9579
Time at the periphery, For female mice	Fig. 5D	p(ABA)=0.4254	**p($TLR4^{IEC}$)=0.0208**	p(Int)=0.3433
Immobility time, For male mice	Fig. 5E	**p(ABA)=0.0009**	p($TLR4^{IEC}$)=0.5351	p(Int)=0.2730
Immobility time, For female mice	Fig. 5F	p(ABA)=0.0513	p($TLR4^{IEC}$)=0.3364	p(Int)=0.8333
<u>Corticosterone</u>				
For male mice	Fig. 5G	**p(ABA)=0.0049**	p($TLR4^{IEC}$)=0.1490	p(Int)=0.5838
For female mice	Fig. 5H	p(ABA)=0.0646	p($TLR4^{IEC}$)=0.1977	p(Int)=0.1967
<u>NPY mRNA</u>				
For male mice	Fig. 6A	**p(ABA)=0.0265**	p($TLR4^{IEC}$)=0.5276	p(Int)=0.4474
For female mice	Fig. 6B	p(ABA)=0.4113	p($TLR4^{IEC}$)=0.8826	p(Int)=0.6416
<u>POMC mRNA</u>				
For male mice	Fig. 6C	**p(ABA)=0.0042**	p($TLR4^{IEC}$)=0.3887	p(Int)=0.4510
For female mice	Fig. 6D	p(ABA)=0.2935	p($TLR4^{IEC}$)=0.5767	p(Int)=0.3736
<u>MC4R mRNA</u>				
For male mice	Fig. 6C	**p(ABA)=0.0014**	p($TLR4^{IEC}$)=0.1801	p(Int)=0.6006
For female mice	Fig. 6D	p(ABA)=0.8269	p($TLR4^{IEC}$)=0.5964	p(Int)=0.5562
<u>Total wheel activity per day</u>				
For male mice	Fig. S5A	p(Time)=0.0744	p($TLR4^{IEC}$)=0.8539	p(Int)=0.7764
For female mice	Fig. S5B	p(Time)=0.1777	p($TLR4^{IEC}$)=0.8596	p(Int)=0.8883

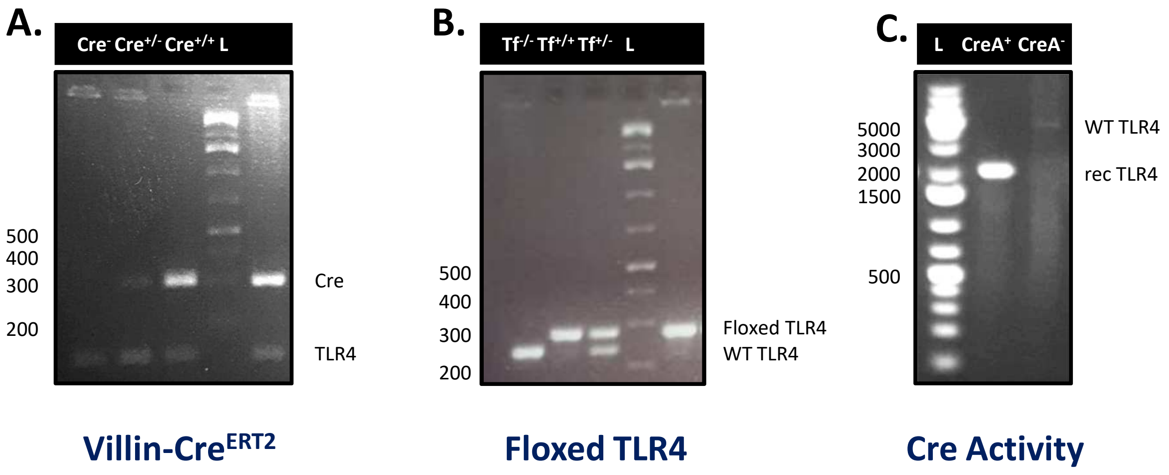

Supplemental Figure S1: Mouse genotyping PCR

(A.) PCRs directed against Villin-Cre^{ERT2} sequence allow to confirm the presence of the Cre recombinase gene (Cre) while the amplification of TLR4 DNA (TLR4) is a positive control of PCR to avoid false negatives. (B.) The Floxed DNA (Floxed TLR4) amplification reveals the presence or not of the first LoxP site in TLR4 gene or of wild-type (WT) allele. Tf$^{+/+}$, Tf$^{+/-}$ or Tf$^{-/-}$ indicate the homozygous, heterozygous or WT mice, respectively. For both Villin-Cre^{ERT2} and Floxed TLR4, mice were genotyped on juvenile samples. (C.) To confirm the first genotype and the Cre Activity in the intestine, *post mortem* genotyping was also performed. The Cre Activity PCR amplifies recombinant DNA by using primers from each side of LoxP sites (rec TLR4). In the absence of Cre Activity, only WT TLR4 gene was amplified.

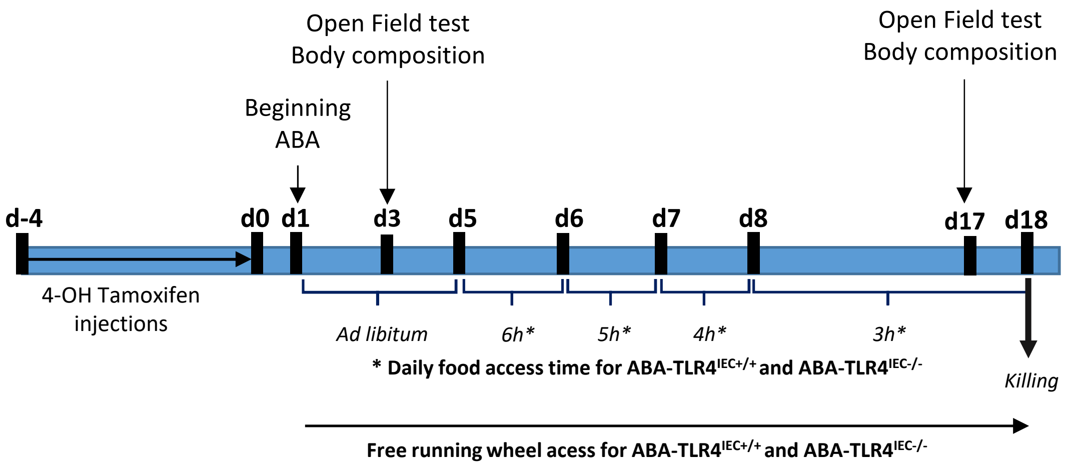

Supplemental Figure S2: **Timeline of experimental protocol**

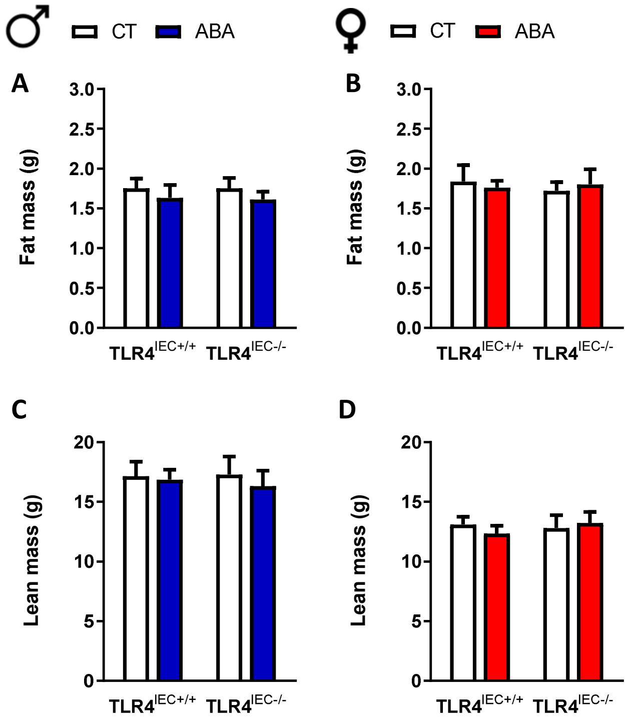

Supplemental Figure S3. Body composition at day 3 in male and female mice. Fat (**A-B**) and lean mass (**C-D**) in male (A-C) and female (B-D) mice between TLR4$^{IEC+/+}$ and TLR4$^{IEC-/-}$ measured at day 3 of the ABA procedure (blue and red bars in male and female mice, respectively).

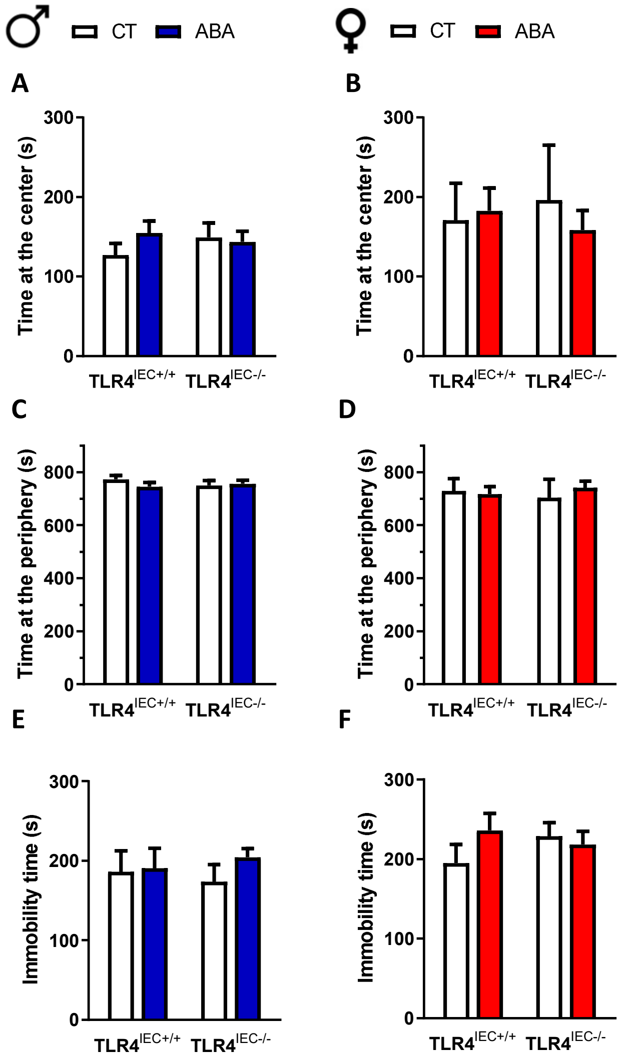

Supplemental Figure S4. Open field test at d3 in male and female mice. Time at the centre (**A**), the periphery (**C**) and immobility time (**E**) during open field test at d3 inTLR4$^{IEC+/+}$ and TLR4$^{IEC-/-}$ CT (open bars) and ABA (blue bars) male mice. Time at the centre (**B**), the periphery (**D**) and immobility time (**F**) during open field test at d3 in TLR4$^{IEC+/+}$ and TLR4$^{IEC-/-}$ CT (open bars) and ABA (reds bars) female mice.

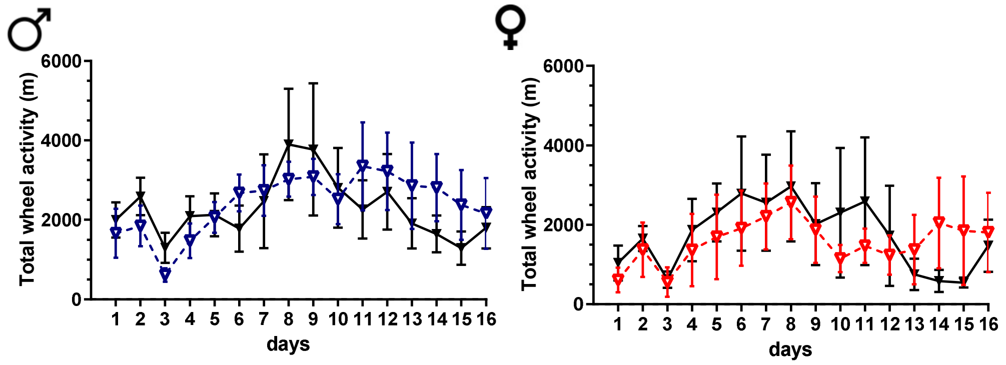

Supplemental Figure S5. Total wheel activity in male and female ABA mice. Total wheel activity measured in male (**A**) and female (**B**) mice in response to the ABA model. Mice were specifically invalidated for TLR4 in in intestinal epithelial cells (TLR4$^{IEC-/-}$, open triangles) or not (TLR4$^{IEC+/+}$, closed triangles).
